# Financial Network Analysis on the Performance of Companies Using Integrated Entropy–DEMATEL–TOPSIS Model

**DOI:** 10.3390/e24081056

**Published:** 2022-07-31

**Authors:** Kah Fai Liew, Weng Siew Lam, Weng Hoe Lam

**Affiliations:** Department of Physical and Mathematical Science, Faculty of Science, Universiti Tunku Abdul Rahman, Kampar Campus, Jalan Universiti, Bandar Barat, Kampar 31900, Perak, Malaysia; liewkf@utar.edu.my (K.F.L.); lamws@utar.edu.my (W.S.L.)

**Keywords:** causal relationship, entropy, TOPSIS, DEMATEL, multi-criteria decision making, financial ratio

## Abstract

In this paper, we propose a multi-criteria decision making (MCDM) model by integrating the entropy–DEMATEL with TOPSIS model to analyze the causal relationship of financial ratios towards the financial performance of the companies. The proposed model is illustrated using the financial data of the companies of Dow Jones Industrial Average (DJIA). The financial network analysis using entropy–DEMATEL shows that the financial ratios such as debt to equity ratio (DER) and return on equity (ROE) are classified into the cause criteria group, whereas current ratio (CR), earnings per share (EPS), return on asset (ROA) and debt to assets ratio (DAR) are categorized into the effect criteria group. The top three most influential financial ratios are ROE, CR and DER. The significance of this paper is to determine the causal relationship of financial network towards the financial performance of the companies with the proposed entropy–DEMATEL–TOPSIS model. The ranking identification of the companies in this study is beneficial to the investors to select the companies with good performance in portfolio investment. The proposed model has been applied and validated in the portfolio investment using a mean-variance model based on the selection of companies with good performance. The results show that the proposed model is able to generate higher mean return than the benchmark DJIA index at minimum risk. However, short sale is not allowed for the applicability of the proposed model in portfolio investment.

## 1. Introduction

Nowadays, a company’s financial performance is of great concern to investors, shareholders and stakeholders. It is important to understand the information about companies’ performance [1]. Aktan and Bulut [1] define a company’s financial performance as its ability to generate new resources from day to day operations over a given time period. Financial performance is crucial to describe how well a company utilizes assets from its business and generates revenue [2]. Moreover, financial performance can also be a measure of a company’s overall financial health over a given time period. Financial stability is quite important to companies, as it enables the companies to utilize their resources optimally, maximize the potential of service to the customers, enhance the ability to pay employees, creditors and vendors on time, as well as the maintenance of good credit risk [2].

A comprehensive study on the financial performance of the company is an essential area of concern that has attracted the attention of organizational managers, investors, public, government and researchers [2]. The financial performance of a company is important because it can identify the fallible areas and improve overall efficiency for the company. Performance evaluation of companies is excellent when used to interpret the past and present financial health of the company, as well as to predict the company’s condition in the future [3]. Financial ratios play an important role as measurement tools to measure the financial performance and financial assets of the companies. According to previous studies [4,5,6,7], financial ratios have been used as indicators to assess companies’ financial performance [8,9,10,11].

Pradesyah and Putri [12] have evaluated the level of profitability ratios and the level of financial performance of Islamic banking by using the financial ratios of return on equity (ROE) and return on asset (ROA). Financial ratios such as debt to equity ratio (DER), current ratio (CR), ROE and ROA are considered by Ali and Faisal [13] to investigate the financial performance of the business organization in Saudi Arabia. Delvia and Alexander [14] have used the debt to assets ratio (DAR), CR and ROA to measure the financial performance of the companies that are listed on the Indonesia Stock Exchange. Otekunrin et al. [15] have carried out an analysis on the performance of companies by taking the financial ratios into consideration. These financial ratios include earnings per share (EPS), ROA, ROE, CR and DAR, and play a central role in assessing companies’ financial performance based on the past studies [16,17,18,19,20]. Thus, it is essential that financial ratios such as EPS, DAR, ROE, CR, ROA and DER are incorporated in this study to evaluate the financial performance of the companies.

Based on the previous studies, the multi-criteria decision making (MCDM) model has been utilized for measuring the companies’ financial performance. Besides, the MCDM model is also capable of identifying the ranking of the companies, and hence the financial performance of the companies can be compared. Yalcin et al. [21] have proposed an MCDM model to determine Turkish manufacturing companies’ financial performance. Furthermore, Chang and Tsai [22] have evaluated the financial performance of wealth management banks with AHP and VIKOR models. The AHP model is utilized to identify the criteria weights, whereas the VIKOR model is employed to rank the seven wealth management banks’ financial performance. Erdogan and Yamaltdinova [23] have assessed the financial performance of thirteen tourism companies using the TOPSIS model.

In this research, the financial ratios’ weights are determined by using the entropy weight method and the DEMATEL model. Entropy is a commonly used method of determining the objective weights [24,25,26,27]. With this method, the weight can effectively eliminate human interference [28]. Thus, the results obtained from the study are more objective. The entropy method has previously been considered by researchers in various fields, such as firm decision making [29], investment decision making [30], multiple attribute decision making [31,32], landslide susceptibility map [33], risk assessment [34], incomplete information systems [35] and coal-fired power units [36]. The decision makers are able to avoid the subjectivity of weight selection using the entropy method [37]. Therefore, the entropy weight method is introduced to reduce biases by giving the objective weights.

DEMATEL is a model that is used to build and analyze a structural model for analyzing the influence relation among criteria [38]. DEMATEL is a well-known model that is utilized to tackle intertwined and complicated problems [39,40,41]. This model is capable of converting the interrelationship among multiple criteria quantitatively [3]. The DEMATEL model is able to establish the direct-relation matrix [27,39,42,43,44]. The objective of the DEMATEL model is to gather collective knowledge in order to identify the causal relationships among strategic criteria [45,46]. Based on the DEMATEL model, the criteria are categorized into cause factor and effect factor. The DEMATEL model aims to convert the relationship between effect and cause factors into an intelligent structure model of the system [47]. The DEMATEL model has been widely used in various fields such as green supply chain [48], supplier selection [49], safety management decision making [50], construction sites [51], optimal sensor placement [52], financial performance [53,54,55,56] portfolio selection [57], emergency alternative evaluation [58] and optimal public charging infrastructure operators [59]. For the DEMATEL model, the criterion with high prominence will play an important role in the evaluation [60]. In this way, more reasonable comprehensive weights for the criteria can be obtained.

The TOPSIS model is an MCDM method which is used to measure the distance to an idealized solution [25]. The importance of the TOPSIS model is the identification of the ranking of the alternatives based on the performance of the alternative. In the TOPSIS model, the performance of the alternatives is identified based on the ranking. It implies that the most preferred alternative has the closest distance from the positive ideal solution (PIS) and the farthest distance from the negative ideal solution (NIS) [61]. In the recent literature, past studies have shown the robustness and reliability of the TOPSIS model in assessing the performance of the alternatives from different aspects such as public blockchain evaluation [62], green public procurement [63], Coupled Model Intercomparison Project evaluation [64], power industry [26], agricultural water resources management [65], international trade performance [66], sustainable development [67,68] and healthcare [69].

According to our best understanding, there has been no comprehensive study done on the causal relationship of financial ratios towards the financial performance of companies by integrating the entropy–DEMATEL–TOPSIS model. In this study, the entropy weight method and DEMATEL model are proposed to identify the weights of the criteria based on the entropy value that can reflect the amount of information in each criterion. However, the entropy weight method is focused solely on finding the weights of the criteria without considering the interaction between each criterion [70]. Therefore, the DEMATEL model is proposed to overcome this problem by constructing a direct impact matrix that can identify the logical relationship among the criteria [59]. The entropy–DEMATEL model is suitable to be proposed in this study because it can determine the weights as well as the causal relationship between the financial ratios [27]. In this research, the hybridization of the entropy weight method and DEMATEL model is employed to determine the criteria weights. The proposed entropy–DEMATEL–TOPSIS model is able to analyze the causal relationship of financial ratios towards the financial performance of the companies. A case study using the companies or components of the Dow Jones Industrial Average (DJIA) is done to illustrate the proposed model.

The DEMATEL model is used to consider the interaction among the criteria. After that, based on the integration of entropy weight method and DEMATEL model, a TOPSIS model is proposed to determine the ranking of the 30 companies of DJIA. This paper aims to bridge the research gap by proposing an MCDM model, namely, entropy–DEMATEL–TOPSIS model, to analyze the causal relationship of financial ratio towards the financial performance of the companies. Firstly, the weights of the financial ratios are identified with entropy–DEMATEL. Secondly, the causal relationship among the financial ratios is determined using entropy–DEMATEL. Finally, the companies’ financial performance is compared and ranked using the TOPSIS model. The merits of the proposed entropy–DEMATEL–TOPSIS model in this study are that it assists the investors in identifying and selecting the companies with good financial performance for portfolio investment. Hence, the investors are able to determine the optimal portfolio from the companies with good financial performance using the proposed model.

Table 1 presents the recent state of the art on the financial performance evaluation based on different methods.

The rest of the manuscript is organized as follows. The materials and methods of this study are introduced in Section 2. In this section, the research development and methodology of the proposed entropy–DEMATEL–TOPSIS model are discussed. Section 3 presents the empirical results of this study. The last section of this article draws its conclusions.

## 2. Materials and Methods

### 2.1. Research Development

In this paper, we propose an MCDM model, namely, the entropy–DEMATEL–TOPSIS model, to analyze the causal relationship of financial ratios towards the financial performance of the companies of DJIA. The proposed model comprises three major stages, as shown below:

Stage 1: Identify the weights of the decision criteria (financial ratios) with the entropy–DEMATEL model;

Stage 2: Determine the causal relationship among the financial ratios with the entropy–DEMATEL model;

Stage 3: Compare and rank the decision alternatives (companies) with the TOPSIS model.

Table 2 presents the proposed research framework to analyze the causal relationship of financial ratios towards the financial performance of the companies with the integrated entropy–DEMATEL–TOPSIS model.

Table 2 depicts the proposed research framework, which consists of three levels. The top level is the main objective of the study, whereas the middle level is the decision criteria. Lastly, the bottom level is the decision alternatives. The proposed framework is constructed to analyze the causal relationship of financial ratio towards the financial performance of the companies with the integrated entropy–DEMATEL–TOPSIS model. The companies’ financial performance is evaluated by the important financial ratios, namely, EPS, DAR, ROE, CR, ROA and DER according to the past studies [16,17,18,19,20]. These financial ratios are treated as the decision criteria in this study [71,72].

The data of this study is obtained from Bloomberg. A real case study is carried out to assess the financial performance of the companies of DJIA between the period 2015 and 2020 using the proposed model. The companies of DJIA have been studied by past researchers in portfolio investment [73,74,75].

Section 2.2 presents the proposed model’s methodology. The proposed model is applied in the portfolio investment with a real case study on DJIA as described in Section 2.3.

### 2.2. Proposed Entropy–DEMATEL–TOPSIS Model

In the first stage, the financial ratios’ objective weights are identified with the entropy–DEMATEL model. The entropy weight method is suitable to be employed in this study as it is able to avoid the subjectivity of weight selection [76,77,78].

In the second stage, the causal relationship between the financial ratios is identified using the entropy–DEMATEL model. In this study, DEMATEL model is used to identify the degree of interdependence of criteria. Moreover, DEMATEL model also helps to determine the criteria that are influenced by others [51,79].

In the third stage, the performance and ranking of the decision alternatives (companies) are evaluated and compared using the TOPSIS model. The following steps present the entropy–DEMATEL–TOPSIS model.

Step 1: Compute the financial ratios’ weights by using the entropy weight method. Calculate the proportion “*p_ij_*” of the *n*th criterion value of the *m*th alternative.

(1) $$p_{ij}=\frac{x_{ij}}{\sum_{i=1}^{m}x_{ij}},\;i=1,2,\ldots ,m,\;j=1,2,\ldots ,n$$

where $x_{ij}$ denotes the index value of alternative *j* under criterion *i* of the matrix ${\left(x_{ij}\right)}_{m\times n}$.

Step 2: Determine the entropy “*e_j_*” of criterion *n*.

(2) $$e_{j}=-k\sum_{i=1}^{m}p_{ij}\cdot \ln(p_{ij}),\;j=1,2,\ldots ,n$$

where $k=\frac{1}{\ln(m)}$.

Step 3: Calculate the entropy weight “*w_j_*” of criterion *n*.

(3) $$w_{j}=\frac{1-e_{j}}{\sum_{j=1}^{n}\left(1-e_{j}\right)},\;j=1,2,\ldots ,n$$

Step 4: Construct a direct relation matrix.

(4) $$C=\left[\begin{array}{cccc} 0 & w_{12} & \cdots & w_{1n}\\ w_{21} & 0 & \cdots & w_{2n}\\ \vdots & \vdots & \vdots & \vdots \\ w_{n1} & w_{n2} & \cdots & 0 \end{array}\right]$$

where $w_{ij}=\frac{w_{i}}{w_{j}}$ for i,j=1,2,3,…,n.

Step 5: Build a normalized direct relation matrix.

(5) $$\lambda =\frac{1}{\underset{1\leq i\leq n}{\max}(\sum_{j=1}^{n}w_{ij})},\;i,j=1,2,3,\ldots ,n$$

N=λC

Step 6: Obtain the total relation matrix (*T*).

(6) $$T=\underset{k\to \infty }{\lim}(N+N^{2}+N^{3}+\ldots +N^{k})=N{\left(1-N\right)}^{-1}$$

Step 7: Calculate the columns’ sum and rows’ sum in the total relation matrix. $R_{j}$ denotes the sum of the *j*th column, whereas $D_{i}$ denotes the sum of the *i*th row. The indirect and direct influences between the factors are presented with $R_{j}$ and $D_{i}$, respectively.

(7) $$R_{j}=\sum_{i=1}^{n}t_{ij}(j=1,2,3,\ldots ,n)$$

(8) $$D_{i}=\sum_{j=1}^{n}t_{ij}(i=1,2,3,\ldots ,n)$$

Step 8: Calculate the value of (D+R) and (D−R). (D+R) is denoted as “prominence”, which indicates the importance degree of the criterion, while (D−R) is denoted as “relation”, which indicates the extent of the influence. After that, the criteria will be categorized either into cause or effect groups according to their value of (D−R). The criterion is grouped into the effect group if the value of (D−R) is negative. It implies that this criterion is influenced by other criteria. On the other hand, the criterion is grouped into the cause group if the value of (D−R) is positive. It implies that this criterion has a significant impact on other criteria.

Step 9: Formulate the evaluation matrix (${\left(x_{ij}\right)}_{m\times n}$), comprised of *m* alternatives and *n* criteria, where $x_{ij}$ is the index for the decision alternative with respect to each decision criterion.

(9) $${\left(x_{ij}\right)}_{m\times n}=\left[\begin{array}{cccc} x_{11} & x_{12} & \ldots & x_{1n}\\ x_{21} & x_{22} & \ldots & x_{2n}\\ . & & & .\\ . & & & .\\ . & & & .\\ x_{m1} & x_{m2} & \ldots & x_{mn} \end{array}\right]$$

Step 10: Normalize the evaluation matrix.

(10) $$r_{ij}=\frac{x_{ij}}{\sqrt{\sum_{i=1}^{m}x_{ij}^{2}}},\;i=1,2,\ldots ,m,\;j=1,2,\ldots ,n$$

(11) $$R={\left(r_{ij}\right)}_{m\times n}=\left[\begin{array}{cccc} r_{11} & r_{12} & \ldots & r_{1n}\\ r_{21} & r_{22} & \ldots & r_{2n}\\ . & & & .\\ . & & & .\\ . & & & .\\ r_{m1} & r_{m2} & \ldots & r_{mn} \end{array}\right]$$

Step 11: Build the weighting normalization evaluation matrix (*T*).

(12) $$T=\left[\begin{array}{cccc} w_{1}r_{11} & w_{2}r_{12} & \ldots & w_{n}r_{1n}\\ w_{1}r_{21} & w_{2}r_{22} & \ldots & w_{n}r_{2n}\\ . & & & .\\ . & & & .\\ . & & & .\\ w_{1}r_{m1} & w_{2}r_{m2} & \ldots & w_{n}r_{mn} \end{array}\right]$$

where $\sum_{j=1}^{n}w_{j}=1$.

Step 12: Acquire the PIS ($A_{b}$) solution and NIS ($A_{w}$) solution.

(13) $$\begin{array}{l} A_{b}=\{\langle \min(t_{ij}|i=1,2,\ldots ,m)|j\in J_{-}\rangle ,\max(t_{ij}|i=1,2,\ldots ,m)|j\in J_{+}\rangle \}\\ \equiv \{t_{bj}|j=1,2,\ldots ,n\}, \end{array}$$

(14) $$\begin{array}{l} A_{w}=\{\langle \max(t_{ij}|i=1,2,\ldots ,m)|j\in J_{-}\rangle ,\langle \min(t_{ij}|i=1,2,\ldots ,m)|j\in J_{+}\rangle \}\\ \equiv \{t_{wj}|j=1,2,\ldots ,n\}, \end{array}$$

Step 13: Calculate the distance from PIS ($d_{ib}$) and NIS ($d_{iw}$).

(15) $$d_{ib}=\sqrt{\sum_{j=1}^{n}{\left(t_{ij}-t_{bj}\right)}^{2}},i=1,2,\ldots ,m$$

(16) $$d_{iw}=\sqrt{\sum_{j=1}^{n}{\left(t_{ij}-t_{wj}\right)}^{2}},i=1,2,\ldots ,m$$

Step 14: Calculate the relative closeness from the ideal solution ($s_{iw}$).

(17) $$s_{iw}=\frac{d_{iw}}{d_{ib}+d_{iw}},\;0\leq s_{iw}\leq 1,i=1,2,\ldots ,m$$

Based on the alternatives’ performance, each alternative will achieve a value of $s_{iw}$.

Step 15: Identify the decision alternatives’ ranking based on the value of $s_{iw}$. The ranking of the alternatives is arranged in descending order according to the value of $s_{iw}$. If a company has a higher value of $s_{iw}$ it implies that the performance of the company is higher.

### 2.3. Application of the Proposed Model in Portfolio Investment

The proposed model is applied in the portfolio investment with a real case study on DJIA. Based on the ranking of the proposed model, the top 15 companies with good financial performance are selected for portfolio investment using the mean-variance (MV) model. Markowitz [80] introduced the MV model by assuming that the investor is rational in minimizing risks and maximizing returns. Furthermore, the MV model is also important to identify an optimal portfolio that can achieve the expected return at minimum risk [81,82,83,84,85]. The MV model is applied by researchers in portfolio optimization [86,87,88,89,90].

The optimal portfolio is constructed with the MV portfolio optimization model [91]. The formulation of the MV portfolio optimization model is depicted below:

(18) $$\mathrm{Minimize}\;\sum_{i=1}^{n}\sum_{j=1}^{n}\sigma_{ij}x_{i}x_{j}$$

Subject to

(19) $$\sum_{j=1}^{n}r_{j}x_{j}\geq \rho$$

(20) $$\sum_{j=1}^{n}x_{j}=1$$

(21) $$x_{j}\geq 0,\;j=1,2,3,\ldots ,n$$

where

*n* is the number of assets,
$\sigma_{ij}$ is the covariance between assets *i* and *j*,
$x_{j}$ is the weight invested in asset *j*,
$x_{i}$ is the weight invested in asset *i*,
ρ is a parameter representing the target rate of return required by an investor,
$r_{j}$ is the expected return of asset *j* per period.

Equation (18) presents the objective function which minimizes the portfolio risk. Equation (19) is used to obtain the returns at the desired level of return. Equation (20) is used to set the sum of all the assets’ weights equal to one. Equation (21) is to ensure the weights of all the assets are non-negative. Short sale is not allowed for the MV portfolio optimization model, as shown in the Equation (21), which requires positive weights of assets. This is the limitation of the proposed entropy–DEMATEL–TOPSIS integrated into portfolio optimization model.

The portfolio mean return is presented below [92]:

(22) $$r_{p}=\sum_{j=1}^{n}r_{j}x_{j}$$

where

$r_{p}$ is the portfolio mean return,
$x_{j}$ is the weight invested in asset *j*,
$r_{j}$ is the expected return of asset *j* per period.

The portfolio performance ratio’s formula is shown below [92]:

(23) $$\mathrm{Portfolio}\;\mathrm{performance}\;\mathrm{ratio}=\frac{\mathrm{Portfolio}\;\mathrm{mean}\;\mathrm{return}}{\mathrm{Portfolio}\;\mathrm{risk}}$$

## 3. Empirical Results

**Figure 1:**
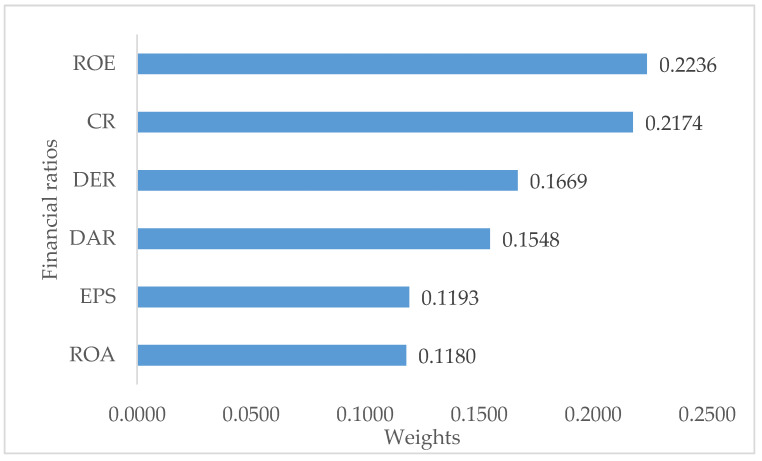
Entropy–DEMATEL weights of financial ratios.

In this paper, the causal relationship of financial ratio towards the financial performance of the companies is analyzed using the proposed entropy–DEMATEL–TOPSIS model. The weights of financial ratios are determined with the entropy–DEMATEL model as displayed in Figure 1.

Based on the proposed model, the entropy–DEMATEL weights of each financial ratio are determined. As shown in Figure 1, ROE is the most influential financial ratio that affects the companies’ financial performance. ROE obtains the largest weight value with 0.2236 among the financial ratios. The second highest weight is obtained by CR. The weight for CR is 0.2174. Next, DER achieves a weight of 0.1669, followed by DAR (0.1548), EPS (0.1193), and lastly ROA (0.1180).

By using the entropy–DEMATEL model, the financial ratios can be categorized either into cause or effect groups. Table 3 shows the value of (D+R) and (D−R) for the financial ratios.

The entropy–DEMATEL model is capable of determining the importance of the financial ratios, as well as the cause and effect relationship among the financial ratios. The value of D+R refers to the degree of relation between each financial ratio with others. For instance, the financial ratios with a higher value of D+R will have more of a relationship with one another. On the other hand, the extent of influence for each financial ratio is referred by the value of D−R. Considering the significance of financial ratios for the evaluation of the companies’ financial performance, ROE is the most important in terms of degree of importance (D+R), followed by CR, DER, DAR, EPS and finally, ROA. By using the entropy–DEMATEL model, the top three most important financial ratios are ROE, CR and DER, with values of 1.2733, 1.2382 and 0.9503, respectively. On the contrary, the least important financial ratios are DAR, EPS and ROA, with values of 0.8813, 0.6794 and 0.6719, respectively. The values of (D−R) for CR, DAR, DER, EPS, ROA and ROE are −1.0797, −0.5976, 0.7026, −0.1120, −0.0355 and 1.1221, respectively. Hence, CR, DAR, EPS and ROA are classified into the effect criteria group, which implies that CR, DAR, EPS and ROA are influenced by other financial ratios. On the other hand, DER and ROE are grouped into the cause criteria group, which indicates DER and ROE have an impact on other financial ratios.

In this study, the financial ratios with positive value of (D−R) will be grouped into the cause group. DER has the lowest value of (D−R) when compared to ROE in the cause group. DER and ROE have the (D−R) values of 0.7026 and 1.1221, respectively. As a result, DER and ROE are classified into the cause criteria group. In contrast, the most significant effect factor is CR, which has the lowest (D−R) value, with −1.0797. This indicates that CR plays a significant role in assessing the companies’ financial performance. Next, DAR is ranked the second lowest degree (−0.5976) among all effect factors. EPS is in the third place (−0.1120). Lastly, ROA has the (D−R) value of −0.0355. Hence, ROA is placed in the lowest ranking among the effect factors.

**Figure 2:**
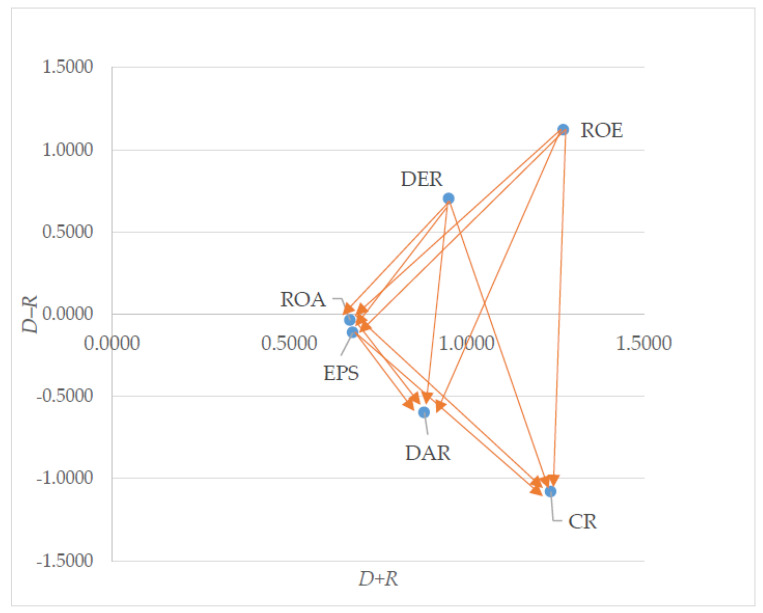
Cause and effect diagram based on entropy–DEMATEL.

Figure 2 shows the cause and effect diagram based on entropy–DEMATEL.

Next, the TOPSIS model is utilized to determine the companies’ financial performance based on the ranking. Table 4 presents the weighting normalization evaluation matrix of the companies by using the entropy–DEMATEL weights with respect to each financial ratio.

According to the weighting normalization evaluation matrix in Table 4, the PIS and NIS for each financial ratio are identified. For PIS, the largest value of ROE, ROA, EPS and CR are selected, while the smallest value of DAR and DER are chosen. On the other hand, for NIS, the biggest value of DAR and DER are opted, whereas the smallest value of CR, EPS, ROE and ROA are chosen.

Table 5 shows the PIS and NIS for each financial ratio using the entropy–DEMATEL weights.

According to Table 5, the PIS for CR, DAR, DER, EPS, ROA and ROE were 0.0839, 0.0050, 0.0024, 0.0609, 0.0466 and 0.2044, respectively. In contrast, the NIS for CR, DAR, DER, EPS, ROA and ROE are 0.0181, 0.0703, 0.1233, 0.0012, 0.0001 and 0.0007, respectively. Next, the distance from the NIS and the distance to the PIS for each company using the entropy–DEMATEL weights are depicted in Table 6.

Table 7 demonstrates the entropy–DEMATEL–TOPSIS scores and ranking of the companies.

As presented in Table 7, BA is identified as the best company in terms of financial performance because BA achieves the highest closeness coefficient, which is 0.5926. Thus, BA obtains the first ranking. This implies that BA has the shortest distance from the PIS and the longest distance from the NIS. On the other hand, NKE achieves the second ranking, with closeness coefficient of 0.4175, followed by AMGN (0.4075), V (0.4064), MSFT (0.4052), INTC (0.4008), TRV (0.4007), CSCO (0.3998), AAPL (0.3975), MMM (0.3965), JNJ (0.3960), UNH (0.3887), HON (0.3838), DIS (0.3827), CVX (0.3776), PG (0.3775), WMT (0.3760), CRM (0.3756), IBM (0.3739), MRK (0.3721), WBA (0.3703), HD (0.3690), DOW (0.3644) and JPM (0.3510). There are a total of six companies with a closeness coefficient which is below 0.3500. These companies consist of VZ (0.3437), AXP (0.3432), KO (0.3406), CAT (0.3353), MCD (0.3335) and, lastly, GS (0.3168). GS achieves the last ranking in terms of financial performance with the lowest closeness coefficient. It is important to determine the companies’ ranking in the financial performance evaluation as it can assist the companies in understanding their financial condition and ranking for the purpose of benchmarking [8]. According to the results, the proposed model has the advantage of determining the financial performance of the companies and providing a reference value for the investors, as well as the shareholders and other stakeholders. The results of this study underscore how the proposed entropy–DEMATEL–TOPSIS model is effective at analyzing the causal relationship of financial ratios towards the financial performance of the companies.

Table 8 presents the summary statistics of the optimal MV portfolio based on the selection of companies with good financial performance using the proposed model.

The summary statistics of the optimal MV portfolio of the proposed model are presented in Table 8. The proposed model generates 0.0125 portfolio mean return at the portfolio risk 0.0375. The portfolio performance ratio obtained in this study is 0.3347. As shown in Table 8, the optimal MV portfolio of proposed model is able to generate higher mean return (0.0125) than the benchmark DJIA index (0.0096). This implies that the proposed model is able to generate the optimal MV portfolio which outperforms the benchmark DJIA index based on the selection of companies with good financial performance. This study is beneficial to the investors, as the outcomes of this study can be served as a reference in portfolio investment.

## 4. Conclusions

In this paper, an MCDM model which integrates the merits of the entropy weight method, DEMATEL and TOPSIS model, is proposed and established to analyze the causal relationship of financial ratios towards the financial performance of the companies. The determination of the weights of financial ratios is resolved by using the concept of entropy. The top three influential financial ratios are ROE, CR and DER based on the proposed model. The results of this study show that DER and ROE are cause factors, whereas the effect factors include CR, DAR, EPS and ROA. Furthermore, for the ranking of the companies, BA is identified as the best company, followed by NKE, AMGN, V and MSFT. The proposed entropy–DEMATEL–TOPSIS model brings a significant impact on the determination of the companies’ ranking, as the integration of entropy weight method, DEMATEL and TOPSIS model helps to analyze the causal relationship of financial ratios towards the financial performance of the companies.

The findings of this research have presented the applicability of the proposed entropy–DEMATEL–TOPSIS model in portfolio investment. The proposed entropy–DEMATEL–TOPSIS model can provide more reliable information that is beneficial to the investors in making better decisions. In this study, the advantages of the proposed entropy–DEMATEL–TOPSIS model are in terms of determining the ranking of the companies and identifying the companies with good financial performance for portfolio investment. The proposed model helps the investors to determine the optimal portfolio from the companies with good financial performance. The optimal MV portfolio of the proposed model is able to generate higher mean return than the benchmark DJIA index. This indicates that the proposed model is able to generate the optimal MV portfolio which outperforms the benchmark DJIA index based on the selection of companies with good financial performance.

For future research, the proposed entropy–DEMATEL–TOPSIS model can be applied to other stock market indices for portfolio investment. The proposed model is able to determine the companies with good financial performance based on the ranking. This will help the investors to select the companies with good financial performance prior to constructing the optimal portfolio for stock market investment. However, short sale is not allowed for the applicability of the proposed model in portfolio investment.

## Figures and Tables

**Table 1:** Recent state of the art on the financial performance evaluation based on different methods.

Description	Field of Study	Method
Evaluate the financial performance of Islamic banks [12]	Bank	Least square method
Investigate the financial performance of the business organization [13]	Business organization	Financial ratio analysis
Measure the financial performance of the listed companies [14]	Listed companies in Indonesia Stock Exchange	Multiple regression analysis
Analyze the financial performance of agriculture and agro-allied firms [15]	Agriculture and agro-allied firms	Multiple regression analysis
Examine the financial performance of agricultural cooperatives [16]	Agricultural cooperatives	Regression analysis
Assess the financial performance of the listed companies [17]	Listed companies in the New York Stock Exchange	Financial ratios analysis and linguistic analysis
Measure the financial performance of oil and gas industry [18]	Oil and gas industry	Financial ratio analysis
Analyze the financial performance of the automotive companies [19]	Automotive companies	Multiple regression analysis
Assess the financial performance of banks [20]	Bank	Panel data regression analysis
Evaluate the financial performance of manufacturing industries [21]	Manufacturing industries	Fuzzy AHP–VIKOR, Fuzzy AHP–TOPSIS
Evaluate the financial performance of wealth management banks [22]	Bank	AHP–VIKOR
Investigate the financial performance of tourism companies [23]	Tourism companies	TOPSIS
**Our study: Analyze the causal relationship of financial ratios towards the financial performance of the companies for portfolio investment.**	**Listed companies of DJIA (Integration of the proposed model** **in portfolio investment)**	**Integration of Entropy–DEMATEL–TOPSIS model in portfolio optimization**

**Table 2:** Proposed research framework.

Level	
Objective	Analysis on the Causal Relationship of Financial Ratios towards the Financial Performance of the Companies
Decision Criteria	Earnings per share (EPS)
(Financial Ratios)	Debt to assets ratio (DAR)
	Return on equity (ROE)
	Current ratio (CR)
	Return on asset (ROA)
	Debt to equity ratio (DER)
Decision Alternatives	3M (MMM)
(Companies)	American Express (AXP)
	Amgen (AMGN)
	Apple (AAPL)
	Boeing (BA)
	Caterpillar (CAT)
	Chevron (CVX)
	Cisco (CSCO)
	Coca-Cola (KO)
	Dow (DOW)
	Goldman Sachs (GS)
	Home Depot (HD)
	Honeywell (HON)
	IBM (IBM)
	Intel (INTC)
	Johnson & Johnson (JNJ)
	JPMorgan Chase (JPM)
	McDonald’s (MCD)
	Merck (MRK)
	Microsoft (MSFT)
	Nike (NKE)
	Procter & Gamble (PG)
	Salesforce (CRM)
	Travelers (TRV)
	UnitedHealth (UNH)
	Verizon (VZ)
	Visa (V)
	Walgreens Boots Alliance (WBA)
	Walmart (WMT)
	Disney (DIS)

**Table 3:** (D+R), (D−R) and the categorization for the financial ratios.

Financial Ratios	D+R	D−R	
CR	1.2382	−1.0797	Effect
DAR	0.8813	−0.5976	Effect
DER	0.9503	0.7026	Cause
EPS	0.6794	−0.1120	Effect
ROA	0.6719	−0.0355	Effect
ROE	1.2733	1.1221	Cause

**Table 4:** Weighting normalization evaluation matrix of the companies by using the entropy–DEMATEL weights with respect to the financial ratios.

Company	EPS	DAR	ROE	CR	ROA	DER
MMM	0.0280	0.0313	0.0192	0.0438	0.0317	0.0130
AXP	0.0185	0.0249	0.0098	0.0285	0.0066	0.0245
AMGN	0.0334	0.0384	0.0176	0.0839	0.0228	0.0202
AAPL	0.0089	0.0241	0.0210	0.0325	0.0383	0.0096
BA	0.0141	0.0143	0.2044	0.0304	0.0076	0.1233
CAT	0.0178	0.0382	0.0090	0.0344	0.0091	0.0240
CVX	0.0075	0.0126	0.0012	0.0285	0.0038	0.0025
CSCO	0.0062	0.0183	0.0077	0.0625	0.0187	0.0048
KO	0.0049	0.0415	0.0131	0.0275	0.0170	0.0197
DOW	0.0054	0.0230	0.0007	0.0487	0.0001	0.0106
GS	0.0609	0.0383	0.0039	0.0277	0.0019	0.0476
HD	0.0244	0.0464	0.0627	0.0306	0.0466	0.0678
HON	0.0215	0.0235	0.0112	0.0334	0.0202	0.0087
IBM	0.0324	0.0280	0.0234	0.0296	0.0172	0.0234
INTC	0.0116	0.0177	0.0093	0.0462	0.0298	0.0036
JNJ	0.0161	0.0153	0.0086	0.0418	0.0206	0.0043
JPM	0.0262	0.0186	0.0049	0.0278	0.0026	0.0221
MCD	0.0214	0.0703	0.0191	0.0412	0.0328	0.0316
MRK	0.0071	0.0239	0.0078	0.0338	0.0148	0.0081
MSFT	0.0113	0.0237	0.0120	0.0670	0.0253	0.0075
NKE	0.0067	0.0140	0.0128	0.0639	0.0342	0.0046
PG	0.0124	0.0208	0.0077	0.0226	0.0188	0.0055
CRM	0.0012	0.0093	0.0007	0.0235	0.0021	0.0024
TRV	0.0325	0.0050	0.0047	0.0327	0.0061	0.0025
UNH	0.0375	0.0203	0.0095	0.0181	0.0175	0.0068
VZ	0.0153	0.0361	0.0265	0.0232	0.0170	0.0303
V	0.0141	0.0173	0.0128	0.0451	0.0326	0.0042
WBA	0.0120	0.0215	0.0057	0.0250	0.0131	0.0074
WMT	0.0138	0.0191	0.0069	0.0214	0.0143	0.0057
DIS	0.0166	0.0194	0.0072	0.0251	0.0190	0.0044

**Table 5:** The PIS and NIS for each financial ratio using the entropy–DEMATEL weights.

Financial Ratios	PIS	NIS
CR	0.0839	0.0181
DAR	0.0050	0.0703
DER	0.0024	0.1233
EPS	0.0609	0.0012
ROA	0.0466	0.0001
ROE	0.2044	0.0007

**Table 6:** The distance from the NIS and the distance to the PIS for each company using the entropy–DEMATEL weights.

Company	Distance from the NIS	Distance from the PIS
MMM	0.1281	0.1950
AXP	0.1111	0.2127
AMGN	0.1335	0.1940
AAPL	0.1311	0.1987
BA	0.2122	0.1458
CAT	0.1076	0.2133
CVX	0.1345	0.2216
CSCO	0.1383	0.2076
KO	0.1100	0.2131
DOW	0.1261	0.2199
GS	0.1021	0.2202
HD	0.1017	0.1739
HON	0.1284	0.2062
IBM	0.1170	0.1958
INTC	0.1377	0.2058
JNJ	0.1358	0.2072
JPM	0.1168	0.2160
MCD	0.1038	0.2074
MRK	0.1264	0.2132
MSFT	0.1373	0.2015
NKE	0.1439	0.2007
PG	0.1299	0.2141
CRM	0.1355	0.2252
TRV	0.1418	0.2120
UNH	0.1333	0.2097
VZ	0.1048	0.2001
V	0.1382	0.2019
WBA	0.1272	0.2162
WMT	0.1298	0.2154
DIS	0.1320	0.2128

**Table 7:** The entropy–DEMATEL–TOPSIS scores ($s_{iw}$) and ranking of the companies.

Company	$s_{iw}$	Ranking
MMM	0.3965	10
AXP	0.3432	26
AMGN	0.4075	3
AAPL	0.3975	9
BA	0.5926	1
CAT	0.3353	28
CVX	0.3776	15
CSCO	0.3998	8
KO	0.3406	27
DOW	0.3644	23
GS	0.3168	30
HD	0.3690	22
HON	0.3838	13
IBM	0.3739	19
INTC	0.4008	6
JNJ	0.3960	11
JPM	0.3510	24
MCD	0.3335	29
MRK	0.3721	20
MSFT	0.4052	5
NKE	0.4175	2
PG	0.3775	16
CRM	0.3756	18
TRV	0.4007	7
UNH	0.3887	12
VZ	0.3437	25
V	0.4064	4
WBA	0.3703	21
WMT	0.3760	17
DIS	0.3827	14

**Table 8:** Summary statistics of the optimal MV portfolio for the period of 2015–2020.

Optimal Portfolio	Value
Portfolio mean return	0.0125
Portfolio risk	0.0375
Portfolio performance ratio	0.3347
DJIA index return (Benchmark)	0.0096
